# Assessment of Public Knowledge about Chronic Kidney Disease and Factors Influencing Knowledge Levels: A Cross-Sectional Study

**DOI:** 10.3390/medicina59122072

**Published:** 2023-11-24

**Authors:** Mansour A. Mahmoud, Alnada Ibrahim, Haifa Abdulrahman Fadil, Ali Mohammed Alalawi, Faris S. Alnezary, Yaser Alahmadi, Sultan Othman Alolayan, Sultan S. Althaqfan, Safaa Omer, Hind Khalid Goresh, Eman Shoroq, Rawan Alghamdi

**Affiliations:** 1 Department of Pharmacy Practice, College of Pharmacy, Taibah University, Madinah 41477, Saudi Arabia; hfadil@taibahu.edu.sa (H.A.F.); fnezary@taibahu.edu.sa (F.S.A.); yahmadi@taibahu.edu.sa (Y.A.); solayan@taibahu.edu.sa (S.O.A.); salthagfan@gmail.com (S.S.A.); 2 Department of Pharmacy Practice, College of Pharmacy, Princess Nourah bint Abdulrahman University, P.O. Box 84428, Riyadh 11671, Saudi Arabia; raalghamdi@pnu.edu.sa; 3 Department of Pharmacology and Toxicology, College of Pharmacy, Taibah University, Madinah 42353, Saudi Arabia; aalawi@taibahu.edu.sa; 4 Department of Clinical Biochemistry, College of Medicine, King Khalid University, Abha 62529, Saudi Arabia; shasn@kku.edu.sa; 5 Department of Clinical Pharmacy, College of Dentistry and Pharmacy, Buraydah Colleges, Buraydah 51418, Saudi Arabia; hindgoresh@gmail.com; 6 Department of Clinical Pharmacy, College of Pharmacy, King Khalid University, Abha 62529, Saudi Arabia; eshorog@kku.edu.sa

**Keywords:** chronic kidney disease, cross-sectional study, knowledge

## Abstract

*Background and Objectives*: Chronic kidney disease (CKD) poses a significant risk for end-stage renal disease (ESRD), cardiovascular diseases, and premature death. The study aims to assess CKD knowledge and predictive variables among the general public. *Materials and Methods:* A cross-sectional study was conducted among the general public in Al Medina Al-Munawara, Saudi Arabia, utilizing a 21-item questionnaire-based approach over a 4-month period from January 2023 to April 2023. The developed questionnaire was validated for readability by experts and refined in light of the feedback received from the experts and the final version was prepared. The reliability of the questionnaire was 0.71, which shows an acceptable level of internal consistency. The data analysis was performed using IBM SPSS software (version 25). *Results*: A total of 777 complete surveys were received after applying the exclusion criteria. The study results revealed that the majority of the respondents had poor knowledge 505 (65%), 203 (26.1) had moderate knowledge, and 69 (8.9%) had good knowledge. Gender (*p* = 0.004), age (<0.001), education level (*p* = 0.039), marital status (*p* = 0.003), and occupation (*p* = 0.002) play significant roles in shaping participants’ knowledge levels regarding CKD. Lower odds of good knowledge were associated with females with an OR (95% CI) of 0.448 (0.263–0.764) and intermediate or higher secondary school level of education displayed an OR (95% CI) of 0.39 (0.18–0.89). Higher odds of good knowledge levels were associated with the age group of 18–27 with an OR (95% CI) of 5.077 (1.21–21.38) and being employed with an OR of 3.555 (1.04–12.21). *Conclusions*: A significant proportion of respondents had poor knowledge about CKD. Several demographic factors were associated with CKD knowledge. Further research is needed to explore these knowledge disparities and develop targeted interventions to improve CKD knowledge among the general public.

## 1. Introduction

Chronic kidney disease (CKD) is characterized by a decline in kidney function, indicated by a glomerular filtration rate (GFR) of less than 60 mL/min per 1.73 m^2^ or evidence of kidney damage lasting for at least 3 months [1,2,3]. This condition poses a significant risk for end-stage renal disease (ESRD), cardiovascular diseases, and premature death [3]. Over the years, the global burden of CKD has shown a substantial increase, resulting in more than 500,000 deaths since 1990 [4,5]. Notably, between 2005 and 2013, the global age-standardized mortality rate for CKD surged by approximately 37% [4]. Furthermore, CKD placed a substantial economic burden on countries and healthcare costs [6,7,8,9]. Despite these alarming statistics, CKD has not received the level of global attention it deserves, necessitating effective public health interventions for prevention and management [4]. 

CKD presents a significant health concern in the Kingdom of Saudi Arabia (KSA). In KSA, the age-standardized prevalence of CKD, considering stages 1–2, stage 3, stage 4, and stage 5 (excluding renal replacement therapy), is estimated to be 9892 per 100,000 individuals. This prevalence rate surpasses the estimates for Western Europe (5446 per 100,000) and North America (7919 per 100,000) [10].

Currently, there are more than 20,000 patients undergoing dialysis and more than 10,000 individuals receiving follow-up care after kidney transplantation in KSA [11]. The combined prevalence of renal replacement therapy in the country is estimated to be 294.3 per million people [11]. Despite the increasing prevalence of CKD, studies have shown that public knowledge about CKD is generally poor [12,13].

The timely identification and treatment of CKD during its early stages can play a crucial role in preventing or delaying disease progression [14]. Numerous clinical practice guidelines, such as those of the National Kidney Foundation, recommend screening individuals with risk factors for CKD [15,16]. Consequently, various screening programs have been implemented worldwide to detect individuals in the community who may have early stages of CKD [17,18].

Evaluating the public’s understanding of CKD holds significant importance in shaping education campaigns for medical professionals, researchers, and healthcare organizations. Nevertheless, there is a noticeable absence of research focused on assessing public awareness of CKD, particularly within the western region of Saudi Arabia, and specifically using validated questionnaires. As a result, the primary goal of this research is to assess the knowledge of a convenient sample of individuals residing in Al Madinah Al Munawara, Saudi Arabia regarding CKD. To achieve this, a newly developed and validated questionnaire was utilized. Furthermore, the study aims to uncover potential predictors that may influence CKD knowledge among the study population.

## 2. Materials and Methods

### 2.1. Study Design and Participants

A cross-sectional study was conducted among the general public in Al Medina Al-Munawara, Saudi Arabia, utilizing a questionnaire-based approach. The Google Form^®^ (**2022**) survey link was distributed via WhatsApp (2023 © WhatsApp LLC) messages to participants who were 18 years and older and could read and understand the Arabic language. To prevent multiple responses from the same participant, respondents were restricted to providing only one response. The study was conducted over a period of four months from January 2023 to April 2023.

### 2.2. Sample and Sampling Technique

A minimum sample size of 385 was calculated with the Raosoft^®^ (© 2004 by Raosoft, Inc., Seattle, WA, USA) online calculator [19] using the following parameters: 5% margin of error, 95% confidence interval for a target population of 1.5 million, and an estimated response distribution of 50%. An extra 20% was added to manage any dropout, and the minimum required sample size was estimated at 462 participants.

### 2.3. Survey Instrument Development and Validation

The 21-item questionnaire was originally developed and validated in Arabic, eliminating the need for translation into English. These items aim to assess the general public’s current knowledge of CKD. The questionnaire items were adapted and modified from previous studies [12,13,20,21] and underwent evaluation for content validity. Four faculty members experienced in pharmacy practice-based research and survey instrument development reviewed the questionnaire for item adequacy, comprehensiveness, type, flow, and clarity. Based on the feedback received from the experts, appropriate changes were made to the questionnaire through an iterative process involving the research team members. These changes included reducing the number of knowledge items from 25 to 21 and converting short-answer questions to multiple-choice questions. Subsequently, the revised version of the questionnaire, deemed suitable for the study, underwent face validity testing. This testing aimed to evaluate the clarity, understanding, readability, ambiguity of items, and questionnaire completion time. Pre-testing was conducted among a sample of seven individuals from the public, and their feedback was taken into consideration. Minor adjustments, such as rewording and paraphrasing certain questions, were made based on the feedback received during the pilot phase. Overall, the research investigators performed several revisions to the originally adapted survey through an extensive iterative process. These revisions addressed the issues identified during the validation process and ensured the questionnaire’s appropriateness for the study. In addition to the 21-item questionnaire, we also collected demographic variables such as age, gender, education status, marital status, occupation, nationality, and smoking status.

### 2.4. Knowledge Assessment

Each knowledge response was scored as “1” (correct) and “0” (wrong), with scores ranging from 0 to 21. Participants’ overall knowledge scores were categorized using a modified Bloom’s criteria cutoff point as good if the score was between 80% and 100% (17–21), moderate if 60–79% (13–16), and poor if <60% (total score < 13) [22].

### 2.5. Analysis

The data were initially recorded in an Excel spreadsheet and underwent a thorough cleaning process. Descriptive statistics were employed to present the data, and categorical variables were reported as frequencies and percentages. To assess the knowledge level across subgroups such as age, gender, education level, marital status, occupation, nationality, and smoking, Pearson’s chi-squared test and Fisher’s exact test were applied as appropriate. Variables identified as significant in the chi-squared test and in Fisher’s exact test were included in a multivariate logistic regression model with good knowledge as an independent variable. The adjusted OR, with a 95% confidence interval (CI), and *p*-value for each determinant were presented. The significance level was set at *p*-value < 0.05. The statistical analysis was performed using IBM SPSS software version 25^®^.

## 3. Results

Initially, 885 responses were collected. After excluding incomplete surveys, we were left with 777 complete surveys. Among these, 584 participants were females, accounting for 75.2% of the total sample, while 193 participants were males, representing 24.8% of the total sample. The age group of 18–27 years had the highest representation, with 442 participants, comprising 56.9% of the total sample. The participants’ characteristics are displayed in Table 1.

In terms of knowledge of the causes of CKD, the results showed that hypertension was correctly identified as a cause of CKD by 328 participants, representing 42.2% of the respondents. Additionally, diabetes was correctly identified as a cause of CKD by 377 participants, accounting for 48.5% of the respondents. Moreover, the majority of participants, 698 individuals, correctly identified the use of drugs that may harm the kidney as a cause of CKD, representing 89.8% of the respondents. Furthermore, inherited diseases were correctly identified as a cause of CKD by 321 participants, comprising 41.3% of the respondents (Table 2).

Regarding the risk factors for CKD, the results revealed that obesity was correctly identified as a risk factor for CKD by 386 participants, accounting for 49.7% of the respondents. Additionally, smoking was correctly identified as a risk factor for CKD by 409 participants, representing 52.6% of the respondents. Moreover, 226 participants correctly identified heart failure as a risk factor for CKD, comprising 29.1% of the respondents. Furthermore, diabetes was correctly identified as a risk factor for CKD by 407 participants, accounting for 52.4% of the respondents. Additionally, hereditary diseases were correctly identified as a risk factor for CKD by 511 participants, representing 65.8% of the respondents. Furthermore, hypertension was correctly identified as a risk factor for CKD by 341 participants, comprising 43.9% of the respondents. Lastly, advanced age was correctly identified as a risk factor for CKD by 279 participants, accounting for 35.9% of the respondents.

**Figure 1:**
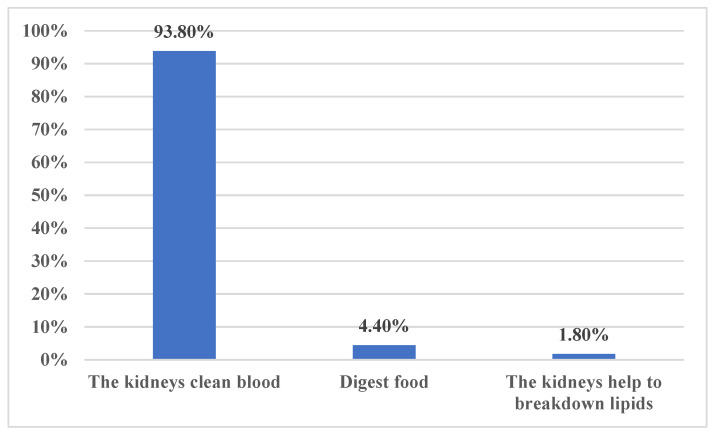
What functions do the kidneys perform in the body?

**Figure 2:**
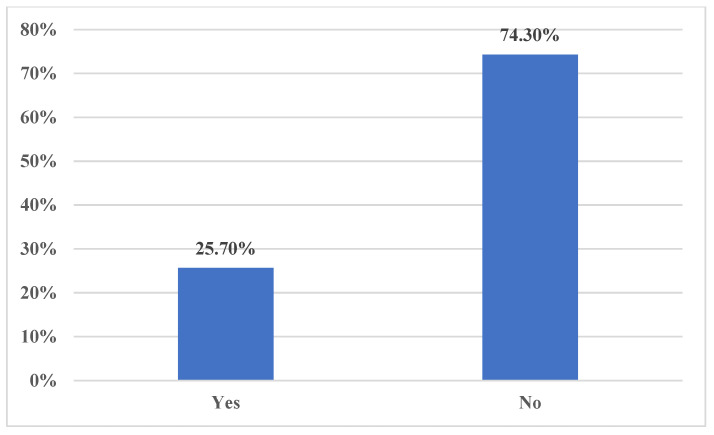
Acute renal failure can be reversable?

In terms of general knowledge about kidney diseases, the majority of participants, 496 individuals (63.8%), correctly responded that a person needs “One” healthy kidney to live a normal life. Furthermore, 729 participants (93.8%) correctly identified that one of the functions performed by the kidneys is cleaning the blood (Figure 1). However, a small number of respondents incorrectly believed that the kidneys help to “Breakdown lipids” (1.8%) or “Digest food” (4.4%) (Figure 1). Regarding treatment options for CKD, the majority of participants, 741 individuals (95.4%), correctly responded that CKD cannot be treated by herbal medicine. Additionally, 641 participants (82.5%) correctly identified kidney transplant as the optimum option for end-stage kidney disease patients. Furthermore, 440 participants (56.6%) correctly identified that end-stage kidney disease can be managed by medications and dialysis. There were some misconceptions among the participants. The majority of participants, 590 individuals (75.9%), incorrectly believed that dialysis is important to manage early CKD. Similarly, 577 participants (74.3%) incorrectly believed that acute renal failure is not reversable (Figure 2). In terms of dialysis, the majority of participants, 276 individuals (35.5%), correctly responded that dialysis can be performed at home, dialysis centers, or hospitals. Moreover, 489 individuals (62.9%) correctly responded that dialysis is not limited to dialysis centers or hospitals only. Additionally, the majority of participants, 774 individuals (99.6%), correctly responded that dialysis cannot be performed at home only.

**Knowledge level across subgroups**

The majority of the respondents had poor knowledge 505 (65%), 203 (26.1%) had moderate knowledge, and only 69 (8.9%) had good knowledge. Significant differences in knowledge levels were found based on various demographic factors. Specifically, there was a statistically significant difference between males and females (*p* = 0.004). Knowledge levels also varied significantly across different age groups (*p* < 0.001). Participants with different educational backgrounds demonstrated a statistically significant difference in knowledge levels (*p* = 0.039). Similarly, there was a significant difference in knowledge levels between single and married participants (*p* = 0.003). Additionally, knowledge levels differed significantly between employed and non-employed participants (*p* = 0.002). These findings collectively demonstrate that gender, age, education level, marital status, and occupation play significant roles in shaping participants’ knowledge levels regarding CKD (Table 3).

**Factors Associated with Knowledge About Kidney Diseases**

Females had lower odds of having good knowledge levels compared to males, with an OR of 0.448 (95% CI: 0.263–0.764). Participants in the age group of 18–27 had significantly higher odds of good knowledge levels, with an odds ratio of 5.077 (95% CI: 1.21–21.38) compared to those aged ≥ 48. Participants with intermediate or higher secondary school education had lower odds of good knowledge levels compared to those with university education, with an OR of 0.39 (95% CI: 0.18–0.89). Employed participants had significantly higher odds of having good knowledge levels compared to non-employed participants, with an odds ratio of 3.555 (95% CI: 1.04–12.21) (Table 4).

## 4. Discussion

Our study revealed concerning gaps in public knowledge about chronic kidney disease (CKD). Only 8.9% of respondents demonstrated good CKD knowledge, while a striking 65% showed poor knowledge. This highlights the need for improved public education around CKD risk factors, symptoms, and prevention.

Several sociodemographic factors emerged as significant influences on knowledge levels. Females and those with intermediate or higher secondary school education had lower odds of possessing good CKD knowledge compared to males and more highly educated participants, respectively. This effect underscores the need to target CKD education efforts towards women and certain education level groups who may have less awareness. On a positive note, higher odds of good knowledge were seen among younger respondents aged 18–27 years and employed individuals. Building on this, future public health campaigns could utilize youth-focused and workplace-based outreach to promote CKD literacy. Overall, our study reveals critical gaps in CKD understanding among the general public in Al Medina Al-Munawara, Saudi Arabia, particularly among key subgroups like women and less educated adults. Tailored education initiatives focused on at-risk demographics are imperative to raise comprehensive CKD awareness and promote prevention.

Our study findings regarding the participants’ knowledge about the causes of CKD can be compared to previous research, providing a broader context for understanding the level of CKD knowledge among different populations. In terms of hypertension and diabetes as leading causes of CKD, our study’s results were slightly lower than those reported by Alobaidi et al. in 2021 [13]. The study conducted by Alobaidi et al. reported higher percentages of participants correctly identifying diabetes (69.2%) and hypertension (54.3%) as causes of CKD compared to our study’s findings of 52.4% and 43.9%, respectively [13]. Albujays et al., 2018 [23] and Ahmed et al., 2018 [24] reported similar results regarding diabetes and hypertension as causes of CKD. These results indicate that a significant proportion of participants in our study incorrectly identified hypertension and diabetes as causes of CKD. However, regarding obesity as a risk factor for CKD, our study’s findings were similar to those of the study conducted by Alobaidi et al. [13], with 49.7% and 51% of participants correctly recognizing obesity as a risk factor, respectively. These results highlight a lack of knowledge and understanding among the study populations regarding the association between these chronic conditions and the development of CKD. Comparable to our study, lower identification of DM and HTN as risk factors for CKD was also reported from Hong Kong [25], Nigeria [26], Singapore [27], and Iran [28]. It is crucial to educate the public about these significant risk factors and emphasize the importance of early identification and management. Increased awareness and understanding of these risk factors can help individuals take proactive measures to prevent or delay the progression of CKD. Early interventions and lifestyle modifications can play a crucial role in preserving kidney function and preventing complications, including ESRD that may require dialysis.

Regarding the question about the number of healthy kidneys needed to live a normal life, studies have consistently shown that the majority of individuals correctly recognize that one functioning kidney is sufficient for a normal life [13,18]. Although about 64% of participants in our study answered this question correctly, several other studies reported higher percentages of individuals with the correct answer. For instance, Alobaidi et al. [13] reported that 80.5% of participants provided the correct response, and an Australian study reported 85.6% [18]. However, a study among primary care patients in Singapore reported that only around 50% of the participants knew the correct answer [27]. These variations in knowledge levels across different populations suggest the need for targeted educational initiatives to improve the understanding of kidney function and the ability to live a normal life with one healthy kidney.

Concerning the impact of age on CKD knowledge, the findings from previous studies have been mixed. While our study found that younger participants had higher odds of having good CKD knowledge levels, other research has shown that older individuals tend to have greater knowledge about CKD. Alobaiadi et al. [13] and Younes et al. [29] found that older age was associated with better CKD knowledge. Conversely, Stanifer et al. found that younger patients had higher knowledge levels about CKD [30]. These discrepancies could be attributed to differences in study populations, healthcare systems, and the prevalence of CKD risk factors across different age groups.

The association between educational attainment and CKD knowledge is well established in the previous literature. Consistent with our findings, several studies have demonstrated that individuals with lower educational levels tend to possess less knowledge about CKD. Younes et al. [28] found that respondents with primary and secondary educational attainment had a significantly lower CKD knowledge score. Gheewala et al. [18] found that respondents with higher educational attainment had a significantly higher CKD knowledge score. Higher education provides individuals with better access to health information, improved health literacy, and a greater ability to understand and apply CKD-related knowledge. By contrast, low educational attainment is associated with adverse outcomes and CKD etiology [31].

While limited research specifically focuses on the association between employment status and CKD knowledge, studies examining the relationship between employment and health knowledge in general have shown that employed individuals may have better access to health promotion programs, occupational health services, and health insurance coverage, which can contribute to higher levels of knowledge about chronic diseases, including CKD. Similar to our study findings, Younes et al. reported that unemployed respondents had a lower CKD knowledge score [29].

Limitations:

While this study provides valuable initial insights into the public knowledge of CKD, some limitations should be considered when interpreting the results. First, the online cross-sectional design and social media-based recruitment may introduce selection bias, as respondents from these platforms may not represent the broader Saudi population. Further research should incorporate more randomized community-based sampling to improve generalizability. Additionally, the self-administered nature of the questionnaire could be subject to recall bias, especially for questions related to personal and family medical history. Incorporating data from medical records could help validate responses in future studies. The questionnaire also assessed knowledge at a single point in time, limiting our ability to infer causal relationships between sociodemographic factors and knowledge levels. Longitudinal studies tracking knowledge over time could better elucidate these associations. Finally, we identified knowledge gaps but did not explore underlying reasons behind them. Qualitative research through interviews and focus groups could provide richer insights into barriers to CKD awareness among subgroups. While an important first step, our findings should be considered preliminary. Follow-up studies addressing these limitations will be essential to derive more conclusive implications to guide targeted educational initiatives and policies aimed at improving the public understanding of this major public health issue.

Recommendations:

Our findings reveal opportunities to improve public knowledge through targeted educational interventions that address the specific gaps identified. For example, inheritance and genetic factors associated with CKD risk were areas of particularly poor understanding. Implementing public health campaigns centered on hereditary kidney diseases could help fill this knowledge deficit.

Healthcare professionals are ideally positioned to lead these CKD education efforts by leveraging their expertise. Physicians can proactively communicate inherited CKD risks to patients with family histories. Pharmacists can provide counseling on medications that may impact kidney health, especially in higher risk groups. Nurses can offer resources on kidney disease screening and prevention behaviors. Multimodal strategies spanning public service announcements, community workshops, social media outreach, and clinic-based education could help reinforce key messages on CKD causes, risks, and prevention across demographics. Collaborations with patient advocacy groups may also widen this reach.

Tailoring health messaging and channels to address specific gaps identified in subgroups will be key to enhancing comprehensive CKD knowledge. With concerted efforts to raise awareness, healthcare professionals have an opportunity to significantly improve the understanding of CKD risk factors in our population.

## 5. Conclusions

Our study’s findings regarding the gender disparity in CKD knowledge, the impact of age, education, and employment on CKD knowledge underscore the need for targeted interventions and educational programs to address the gaps in CKD knowledge, particularly among females and individuals with lower educational attainment. Improving access to healthcare information, promoting health literacy, and implementing inclusive educational initiatives can enhance CKD knowledge and ultimately improve prevention, early detection, and management. While our study provides valuable insights into the specific knowledge items related to CKD, it is important to acknowledge that knowledge levels can vary across different populations and regions. Additional research in diverse settings is warranted to obtain a more comprehensive understanding of CKD knowledge among various demographic groups.

## Figures and Tables

**Table 1:** Demographic characteristics of the participants (*n* = 777).

Characteristic	Frequency	Percent (%)
Gender		
Females	584	75.2
Age		
18–27	442	56.9
28–37	111	14.3
38–47	122	15.7
≥48	102	13.1
Education level		
Intermediate or higher secondary school	162	20.8
University education	615	79.2
Marital status		
Single	449	57.8
Occupation		
Employed	614	79
Nationality		
Saudi	702	90.3
Smoking status		
Smoker	83	10.7
Chronic disease		
Yes	96	12.4

**Table 2:** Percentage of correct response to individual knowledge items (*n* = 777).

Item No		Correct Response N (%)
	What are the causes of CKD?	
1	* Hypertension	328 (42.2)
2	* Diabetes	377 (48.5)
3	* Use of drug that may harm the kidney	698 (89.8)
4	* Inherited diseases	321 (41.3)
	What are the risk factors of CKD?	
5	* Obesity	386 (49.7)
6	* Smoking	409 (52.6)
7	* Heart Failure	226 (29.1)
8	* Diabetes	407 (52.4)
9	* Hereditary diseases	511 (65.8)
10	* Hypertension	341 (43.9)
11	* Advanced age	279 (35.9)
	General Information about kidney diseases	
12	How many healthy kidneys does a person need to live a normal life?	
	* One	496 (63.8)
	Two	281 (36.2)
13	What functions do the kidneys perform in the body?	
	* The kidneys clean blood	729 (93.8)
	The kidneys help to breakdown lipids	14 (1.8)
	Digest food	34 (4.4)
14	CKD can be treated by herbal medicine?	
	Yes	36 (4.6)
	* No	741 (95.4)
15	Kidney transplant is considered the optimum option for end-stage kidney disease patients?	
	* Yes	641 (82.5)
	No	136 (17.5)
16	End-stage kidney disease can be managed by medications and dialysis?	
	* Yes	440 (56.6)
	No	337 (43.4)
17	Dialysis is important to manage early CKD?	
	Yes	590 (75.9)
	* No	187 (24.1)
18	Acute renal failure can be reversable?	
	* Yes	200 (25.7)
	No	577 (74.3)
	Dialysis can be performed at	
19	Home or dialysis centers or hospitals?	
	* Yes	276 (35.5)
	No	501 (64.5)
20	Dialysis centers or hospitals only?	
	Yes	489 (62.9)
	* No	288 (37.1)
21	At home only?	
	Yes	3 (0.4)
	* No	774 (99.6)

* Correct answer.

**Table 3:** Knowledge level across subgroups (*n* = 777).

		Knowledge			
		Good	Moderate	Poor	*p* Value
	Scores	17–21	13–16	<13	
Total	*n* = 777	69 (8.9)	203 (26.1)	505 (65)	
Gender					0.004
	Female	41 (5.3)	151 (19.4)	392 (50.5)	
Age					<0.001
	18–27	56 (7.2)	116 (14.9)	270 (34.7)	
	28–37	3 (0.4)	26 (3.3)	82 (10.6)	
	38–47	7 (0.9)	24 (3.1)	91 (11.7)	
	≥48	3 (0.4)	37 (4.8)	62 (8)	
Education level 3					0.039
	Intermediate or higher secondary school	7 (0.9)	39 (5)	116 (14.9)	
	University education	62 (8)	164 (21.1)	389 (50.1)	
Marital status					0.003
	Single	53 (6.8)	117 (15.1)	279 (35.9)	
Occupation					0.002
	Employed	66 (8.5)	156 (20.1)	392 (50.5)	
Nationality					0.290
	Saudi	66 (8.5)	183 (23.6)	453 (58.3)	
Smoking status					0.891
	Smoker	8 (1%)	20 (2.6)	55 (7.1)	
Chronic disease					0.693
	Yes No	7 (0.9) 62 (8)	28 (3.6) 175 (22.5)	61 (7.9) 444 (57.1)	

Note: Fisher’s exact test, chi-squared test.

**Table 4:** Factors associated with good knowledge about kidney diseases.

Gender		OR (95% Confidence Interval)	*p* Value
	Female	0.448 (0.263–0.764)	0.003
	Male	1	
Age recoded			
	18–27	5.077 (1.21–21.38)	0.027
	28–37	0.864 (0.16–4.51)	0.862
	38–47	1.775 (0.44–7.23)	0.423
	≥48	1	
Education level			
	Intermediate or higher secondary school	0.39 (0.18–0.89)	0.027
	University education	1	
Marital status			
	Single	0.807 (0.32–2.03)	0.649
	Married	1	
Occupation			
	Employed	3.555 (1.04–12.21)	0.044
	Non-employed	1	

## Data Availability

The data presented in this study are available on request from the corresponding author. The data are not publicly available due to Privacy.
